# New lymphatic cell formation is associated with damaged brain tissue clearance after penetrating traumatic brain injury

**DOI:** 10.1038/s41598-021-89616-3

**Published:** 2021-05-13

**Authors:** Fan-Wei Meng, Jun-Tao Yu, Jin-Yuan Chen, Peng-Fei Yang

**Affiliations:** Department of Anatomy and Physiology, Shandong College of Traditional Chinese Medicine, No. 508 Binhaidonglu, Yantai, 264199 Shandong China

**Keywords:** Cell biology, Immunology, Neuroscience, Neurology

## Abstract

We characterized the tissue repair response after penetrating traumatic brain injury (pTBI) in this study. Seventy specific pathogen-free Kunming mice were randomly divided into the following groups: normal control, 1, 3, 7, 15, 21, and 30 days after pTBI. Hematoxylin and eosin (H&E) staining, immunohistochemistry, and immunofluorescence were performed to examine and monitor brain tissue morphology, and the distribution and expression of lymphatic-specific markers lymphatic vessel endothelial receptor-1 (LYVE-1), hematopoietic precursor cluster of differentiation 34 (CD34) antigen, and Prospero-related homeobox-1 (PROX1) protein. H&E staining revealed that damaged and necrotic tissues observed on day 1 at and around the injury site disappeared on day 7, and there was gradual shrinkage and disappearance of the lesion on day 30, suggesting a clearance mechanism. We explored the possibility of lymphangiogenesis causing this clearance as part of the post-injury response. Notably, expression of lymphangiogenesis markers LYVE-1, CD34, and PROX1 was detected in damaged mouse brain tissue but not in normal tissue. Moreover, new lymphatic cells and colocalization of LYVE-1/CD34 and LYVE-1/PROX1 were also observed. Our findings of the formation of new lymphatic cells following pTBI provide preliminary insights into a post-injury clearance mechanism in the brain. Although we showed that lymphatic cells are implicated in brain tissue repair, further research is required to clarify the origin of these cells.

## Introduction

Traumatic brain injury (TBI) can arise from traffic accidents, falls, physical abuse, contact sports, firearm injuries, and natural disasters and is a serious but understated global public health concern^1^. The overall mortality rate was 12.99–17.06% per 100,000 people in China in 2006–2013, of which, males, the elderly, and people living in rural areas had the highest mortality rates^1^. Severe brain trauma can lead to acute swelling and necrosis of brain neurons, disintegration of glial cells, rupture, hemorrhage, contusion and swelling of the cerebral microvascular and subsequent disruption of the cerebral microcirculation, eventually causing permanent neurological defects^2–4^. TBI typically induces blood vessel damage, lipid peroxidation damage, cellular calcium overload, infiltration of leukocytes such as neutrophils and macrophages, and angiogenesis^3^. Due to TBIs being underdiagnosed and the poor efficacy of conventional therapy, patients with moderate to severe conditions or even those with mild conditions, who do not seek prompt or receive proper medical care, may suffer from severe neurological impairment such as long-term coma, paralysis, and cognitive impairment^5^. Given the significant impact of TBI and the limitations of current treatment options, there is an urgent need to discover and develop new technologies for neurological repair and treatment.

The lymphatic system has many functions in normal physiology and pathology, including regulating tissue fluid and blood pressure, alleviating inflammation, lipid absorption, transport and homeostasis, and immune surveillance^6^. During embryonic development, the lymphatic-specific transcription factor Prospero-related homeobox-1 (PROX1) regulates lymphangiogenesis via upregulating the expression of vascular endothelial growth factor receptor 3 (VEGFR-3, a receptor tyrosine kinase) on the membrane of lymphatic endothelial cells^7,8^. Inflammation can activate PROX1 expression via the nuclear factor kappa B (NF-KB) pathway^9^, causing the upregulation of VEGFR-3 expression in lymphatic endothelial cells, stimulating the binding of VEGF-C and VEGF-D, which are released by immune cells during inflammation, and promoting lymphangiogenesis^8–10^. The obstruction of lymphatic drainage can lead to acute edema in inflamed tissues after brain injury leading to neuronal and myelin degeneration as well as scar formation^11,12^, indicating the importance of the lymphatic system in modulating tissue edema and immune response. Thus, blocking inflammation is believed to prevent brain edema and enable the recovery of brain function following TBI.

The discovery of lymphatic vessels in the brain meninges shattered the long-held notion of the lack of lymphatic vessels in the central nervous system^13–16^. Nonetheless, although meningeal lymphatic vessels in the brain have been found, no lymphatic system in the brain parenchyma, composing of neurons and glia, has been discovered to date. Thus, we designed this study to clarify whether a lymphatic system in the brain parenchyma could be involved in alleviating pathophysiological changes and facilitating the repair and regeneration mechanisms after TBI. To characterize the response following penetrating TBI (pTBI) in this study, we examined the expression of lymphatic markers PROX1^17^ and lymphatic vessel endothelial receptor-1 (LYVE-1), as well as hematopoietic precursor cell antigen cluster of differentiation 34 (CD34)^7^ in adult mice, following pTBI. We used H&E staining, immunohistochemistry and immunofluorescence to detect the selected markers in mice. Our findings provide preliminary insights into the mechanism underlying clearance of damaged tissue after pTBI.

## Results

### Pathological changes of brain tissue after pTBI

**Figure 1:**
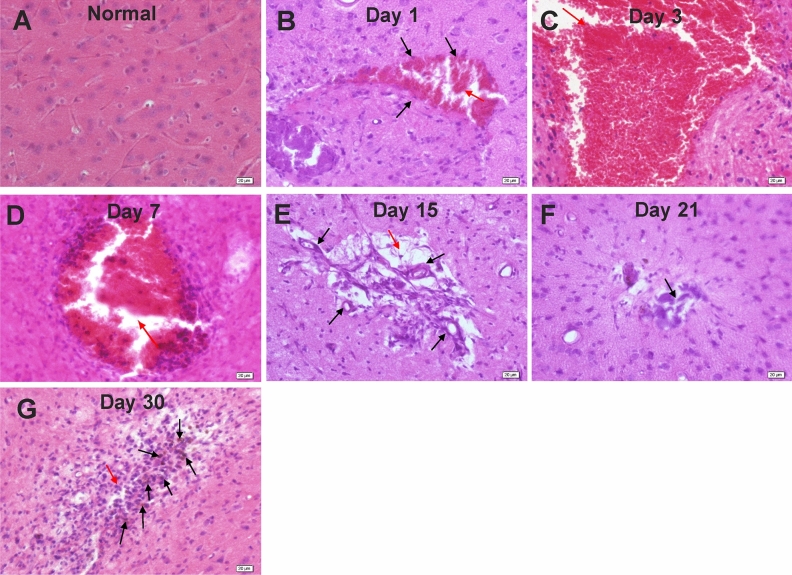
H&E-stained images of (**A**) normal mouse brain tissue and (**B**–**G**) mouse brain tissue on days 1, 3,7, 15, 21 and 30 days, following pTBI. (**A**) Regular size, shape, and arrangement of brain tissue cells were observed for normal mouse brain tissue. (**B**) On day 1 after inflicting pTBI in mouse models, damaged brain tissue and thrombosis (black arrows) were noted at the injury site (lesion indicated with a red arrow). (**C**) The degree of damaged tissue at the injury site (red arrow) was reduced on day 3. (**D**) On day 7 after pTBI, necrotic tissue and thrombus at the injury site (red arrow) were no longer visible. Instead, granulation was observed that was suggestive of new tissues (black arrows). The large lesion at the injury site (red arrow) was still observed. (**E**) The lesion at the injury site (red arrow) was still visible on day 15. (**F**) On day 21, the lesion (red arrow) appeared to have shrunken and hemosiderin particles (black arrows) were observed. (**G**) The lesion was no longer visible on day 30, but some hemosiderin particles remained (black arrows).

We examined the pathological changes of mouse brain tissue morphology following pTBI (Fig. 1). Unlike normal brain tissue (Fig. 1A), the regular arrangement was disrupted following pTBI. Hemorrhage, thrombosis, tissue rupture, and damage could be observed at the injury site on day 1 (Fig. 1B) and less so on day 3 (Fig. 1C). On day 7, necrotic tissue and cells were no longer visible, suggesting clearance, and granulated tissue was observed at the lesion (Fig. 1D). Shrinkage of the lesion was noted on days 15 and 21 (Fig. 1E,F). The lesion was no longer visible on day 30, but a small number of black hemosiderin particles could be seen (Fig. 1G).

### LYVE-1 expression changes in brain tissue after pTBI

**Figure 2:**
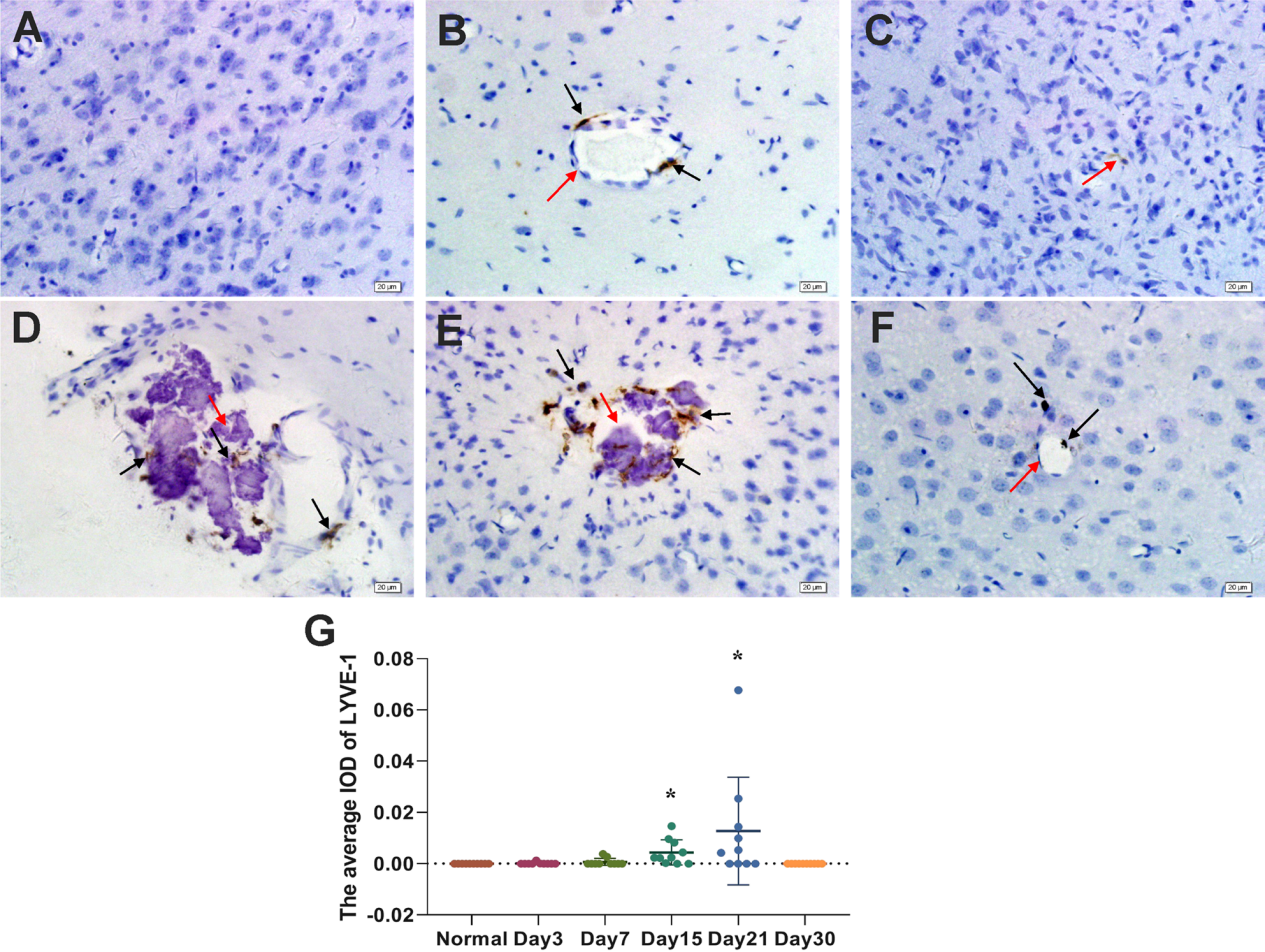
Immunohistochemical staining images showing LYVE-1 expression in (**A**) normal mouse brain tissue and (**B**–**F**) mouse brain tissue on days 3, 7, 15, 21 and 30 days, following pTBI. (**A**) LYVE-1 was not expressed in normal mouse brain tissue. (**B**) LYVE-1-positive (brown; black arrow) vascular endothelial cells became visible around the injury site (lesion indicated with a red arrow) on day 3. (**C**) LYVE-1-positive vascular endothelial cells were clearly visible on day 7. LYVE-1-positive cells (black arrows) were observed at the injury site (red arrow) on days (**D**) 15 and (**E**) 21. (**F**) LYVE-1-positive cells were no longer visible at the injury site (red arrow) on day 30, but black hemosiderin particles (black arrows) were observed. (**G**) Immunohistochemical results of LYVE-1 were analyzed by Image-Pro Plus 6.0 software. The average density (IOD) of LYVE-1-positive cells in normal mouse brain tissue and in mouse brain tissue on days 3, 7, 15, 21 and 30 days, following pTBI. The n = 10/group was used for calculating the average IOD. Comparisons between groups were performed using the Kruskal–Wallis H test followed by Bonferroni post-hoc analysis.

To determine the potential involvement of the lymphatic system in the clearance of damaged and necrotic brain tissue at the injury site, we monitored the expression of LYVE-1, a classical marker of lymphangiogenesis, by vascular endothelial cells using immunohistochemistry. LYVE-1 was not detected in normal brain tissue (Fig. 2A) and could only be detected around the injury site from day 3 following pTBI (Fig. 2B,C). No significant LYVE-1 expression was noted on day 1, thus the data was not shown. LYVE-1-expressing cells were observed at the injury site on day 15 (Fig. 2D) and LYVE-1 expression peaked on day 21 (Fig. 2E). Most of the LYVE-1-positive cells disappeared on day 30, leaving a small number of black hemosiderin particles at the injury site (Fig. 2F). The density (IOD) of LYVE-1-positive cells on days 15 and 21 post-injury was higher than that in the normal group (Fig. 2G; *p* < 0.05). These results indicate the association of lymphangiogenesis with damaged brain tissue clearance.

### CD34 expression changes in brain tissue after pTBI

**Figure 3:**
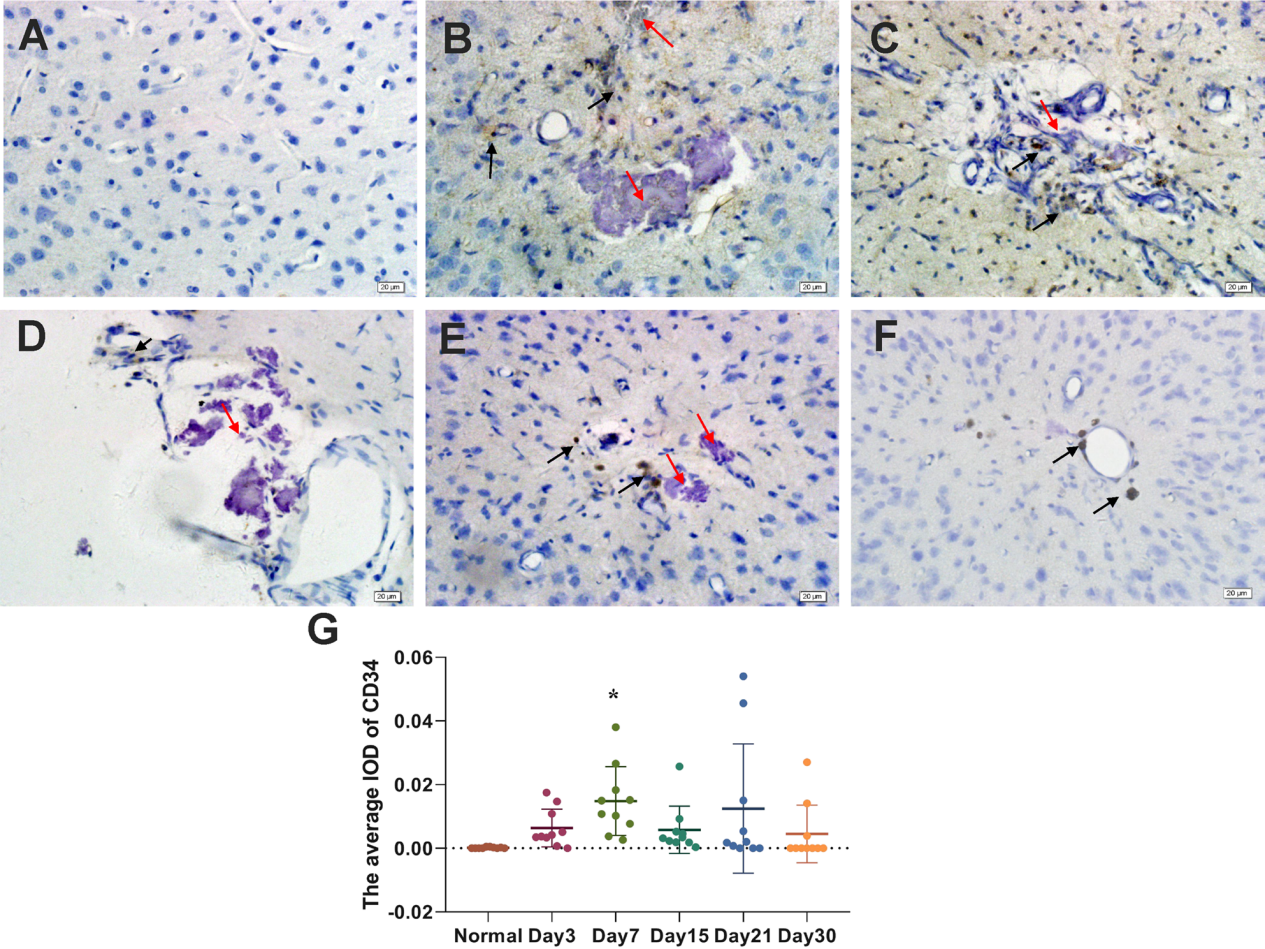
Immunohistochemical staining images showing CD34 expression in (**A**) normal mouse brain tissue and (**B**–**F**) mouse brain tissue on days 3, 7, 15, 21 and 30 days, following the pTBI. (**A**) CD34 was not expressed in normal mouse brain tissue. CD34-positive cells and blood vessels (brown; black arrows) became visible at the injury site (red arrow) on days (**B**) 3 and (**C**) 7. (**D**) After 15 days, CD34 expression at the injury site (red arrow) decreased. (**E**) CD34 expression at the injury site could not be detected on day 21. Instead, black hemosiderin particles (black arrows) were observed at the injury site. (**F**) Some black hemosiderin particles remained visible at the injury site on day 30. (**G**) Immunohistochemical results of CD34 were analyzed by Image-Pro Plus 6.0 software. The average density (IOD) of CD34-positive cells in normal mouse brain tissue and in mouse brain tissue on days 3, 7, 15, 21 and 30 days, following pTBI. The n = 10/group was used for calculating the average IOD. Comparisons between groups were performed using the Kruskal–Wallis H test followed by Bonferroni post-hoc analysis.

Similar to LYVE-1, CD34 expression by vascular endothelial cells was not detected in normal brain tissues (Fig. 3A) but was observed around the injury site from day 3 after pTBI (Fig. 3B). No significant CD34 expression was noted on day 1, thus the data was not shown. Significant CD34 expression peaked on day 7 (Fig. 3C) indicating increased neovascularization around the injury site but decreased on day 15 (Fig. 3D). CD34-expressing cells and blood vessels were not visible on day 21 but some black hemosiderin particles were observed (Fig. 3E). By day 30, although the lesion was no longer visible, some hemosiderin particles at the injury site still remained (Fig. 3F), indicating that recovery was still not complete. The density (IOD) of CD34-positive cells on day 7 post-injury was higher than that in the normal group (Fig. 3G; *p* < 0.05).

### PROX1 expression changes in brain tissue after pTBI

**Figure 4:**
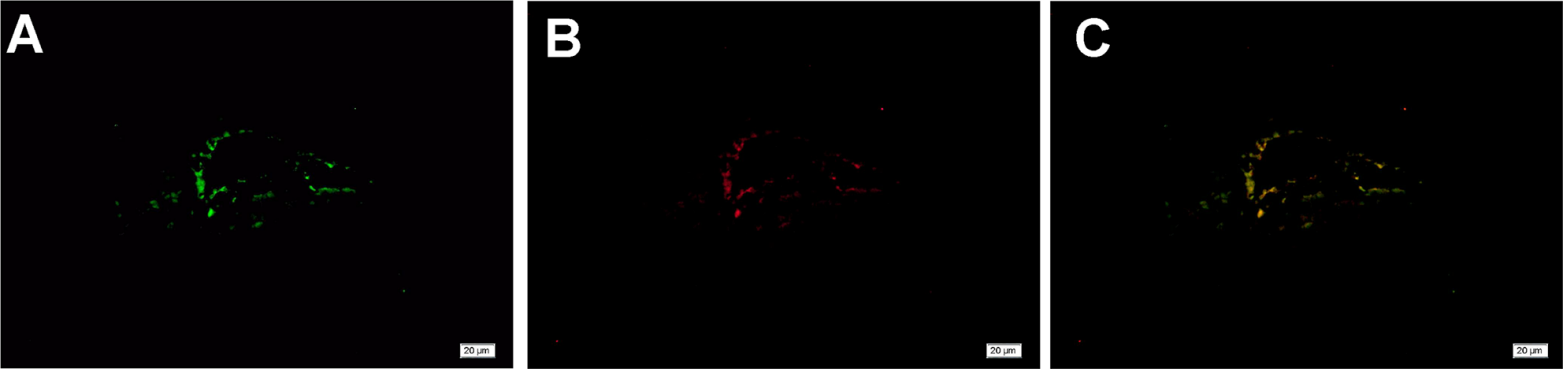
Double immunofluorescence staining images showing CD34/LYVE-1 expression in the brain tissue 3 days after pTBI. Expression of (**A**) LYVE-1 (green); (**B**) CD34 (red); and (**C**) merge (yellow).

**Figure 5:**
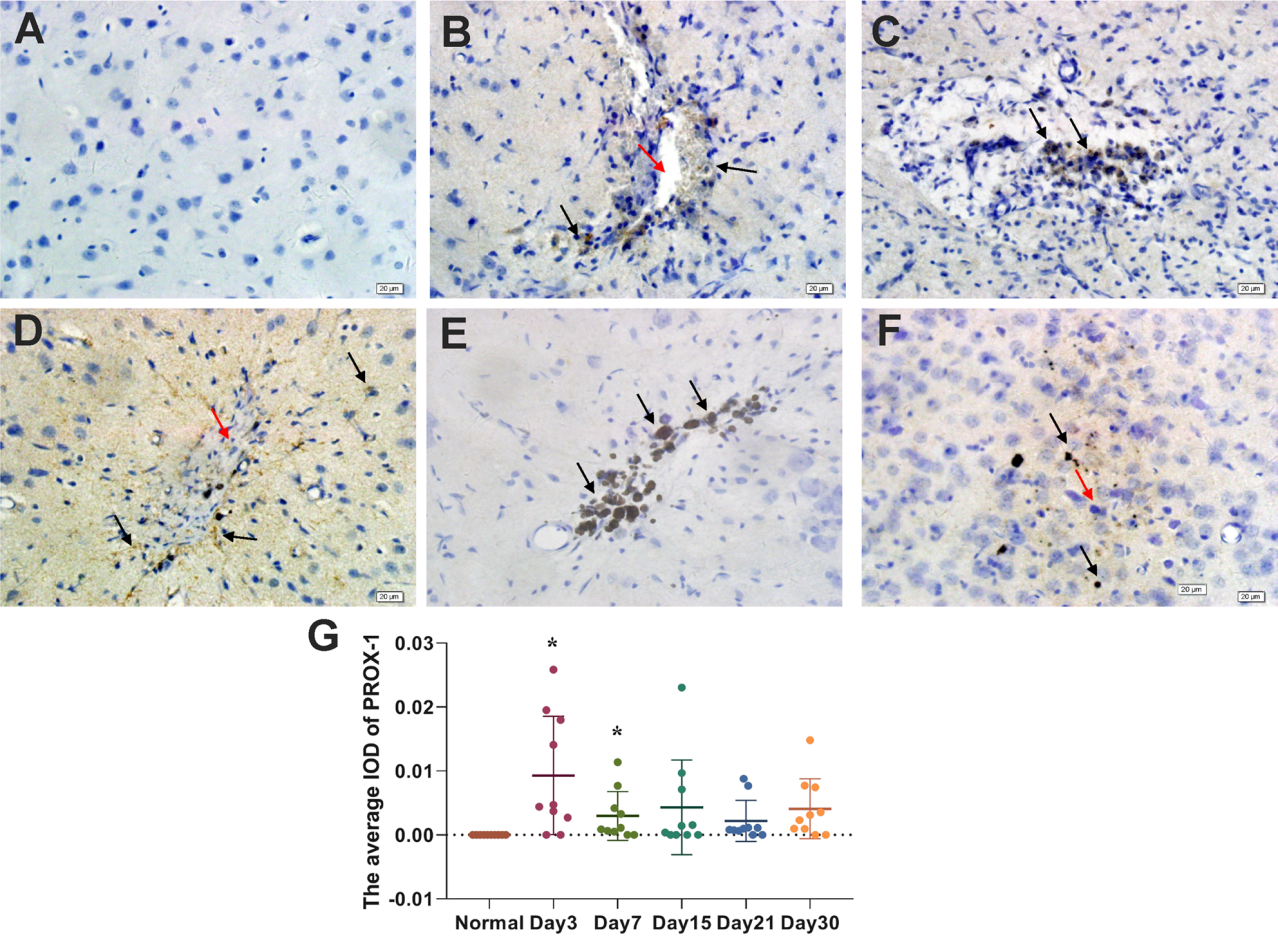
Immunohistochemical staining images showing PROX1 expression in (**A**) normal mouse brain tissue and (**B**–**F**) mouse brain tissue on days 3, 7, 15, 21 and 30 days, following pTBI. (**A**) PROX1 is not expressed in normal mouse brain tissue. PROX1 expression (brown; black arrows) was detected around the injury site (red arrow) on days (**B**) 3 and (**C**) 7. (**D**) PROX1-expressing cells and tubular structures (black arrows) were observed around the injury site on day 15 and a small number of black hemosiderin particles (black arrowheads) were visible. (**E**,**F**) PROX1 expression (black arrows) and black hemosiderin particles (black arrowheads) were still visible at the injury site on days 21 and 30. (**G**) Immunohistochemical results of PROX1 were analyzed by Image-Pro Plus 6.0 software. The average density (IOD) of PROX1-positive cells in normal mouse brain tissue and in mouse brain tissue on days 3, 7, 15, 21 and 30 days, respectively, following pTBI. The n = 10/group was used for calculating the average IOD. Comparisons between groups were performed using the Kruskal–Wallis H test followed by Bonferroni post-hoc analysis.

**Figure 6:**
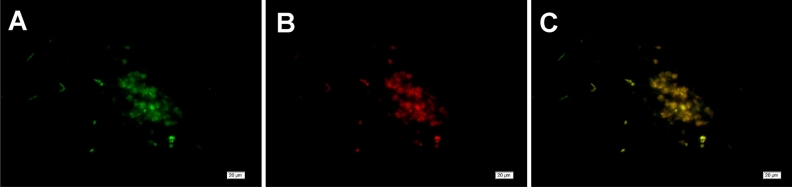
Double immunofluorescence staining images showing LYVE-1/PROX1 expression in brain tissue 7 days after pTBI. Expression of (**A**) LYVE-1 (green); (**B**) PROX1 (red); and (**C**) merge (yellow).

Immunofluorescence revealed the co-expression of LYVE-1 and CD34 around the injury site (Fig. 4A–C). Both LYVE-1 and CD34 were expressed from day 3 after injury (Fig. 4C). PROX1 regulates the differentiation and maturation of lymphatic endothelial cells into either CD34-only or LYVE-1-only expressing differentiated neovascular endothelial cells. Thus, we also assessed PROX1 expression after pTBI. Vascular endothelial cells in normal brain tissues do not express PROX1 (Fig. 5A) but PROX1 expression was detected at and around the injury site from day 3 (Fig. 5B) and could still be detected on day 30 (Fig. 5C–F). No significant PROX1 expression was noted on day 1, thus the data was not shown. Black hemosiderin particles at the injury site were visible on days 15, 21, and 30 (Fig. 5D). Relatively higher PROX1 expression was observed around the injury site on day 3 (Fig. 5G; *p* < 0.05). Colocalization of LYVE-1 and PROX1 expression was similarly observed at and around the injury site at day 7 post-injury, forming a tubular pattern (Fig. 6A–C), suggesting the association of PROX1 with lymphangiogenesis following pTBI. Both LYVE-1 and PROX1 were expressed from day 7 post-injury, albeit at different levels (Fig. 6C).

## Discussion

Lymphangiogenesis is critical for preventing tissue edema and nerve damage following TBI by allowing rapid and timely macrophage recruitment and microglia activation following injury for repair and regeneration^18^. Although meningeal lymphatic vessels have been discovered in the brain^15,16^, the absence of a lymphatic system in the brain parenchyma^19^ raised questions about the repair and recovery mechanism following brain trauma. We speculated that lymphangiogenesis could form part of the post-injury response and promote edema clearance, thereby enabling brain tissue recovery. Notably, we did not observe a lymphatic system in the brain of normal mice but noted the formation of new lymphatic cells in the brain parenchyma following pTBI, suggesting the probable association of lymphangiogenesis with post-pTBI tissue repair and recovery response.

Because PROX1 can modulate VEGFR, the key regulator of lymphangiogenesis, via the NF-kB pathway^9^, we monitored PROX1 expression as a marker of lymphangiogenesis in mouse brain tissue after pTBI. We also monitored the expression of LYVE-1, a well-characterized marker of lymphatic endothelium^7^ in this study. H&E staining showed that the necrotic tissue and lesion at the injury site shrank and disappeared by day 30, indicating clearance of tissue edema and restoration of normal shape and function of brain tissue. Vascular endothelial cells at and around the injury site started expressing CD34, LYVE-1, and PROX1 from day 3 and their expression were colocalized at and around the injury site, suggesting that PROX1 can induce the differentiation of CD34-positive vascular endothelial cells into LYVE-1-positive lymphatic cells following pTBI. Although we did not observe lymphatic vessels, we speculate that these new lymphatic cells might be involved in alleviating brain tissue edema by increasing the return of local tissue fluid and inflammatory cells. Also, these LYVE-1-positive lymphatic cells may have facilitated the removal of necrotic myelin sheath and cells and promoted the formation of new myelin sheath by glial cells, thereby promoting the recovery of brain nerve function. Future studies are needed to validate these speculations.

TBI induces inflammation, wherein growth-inhibiting substances including nitric oxide and proteoglycans are secreted by glial and vascular cells that gather in the damaged area leading to the formation of glial scars^20^. PROX1 is an important mediator of inflammation-induced lymphangiogenesis in adults^7,9^, differentiation of neonatal lymphatic endothelial cells as well as a key marker for the differentiation and maturation of neural stem cells. Thus, its varied roles could explain the lack of correlation between the PROX1 and LYVE-1 expression profile and why PROX1 level remained high at day 30 post-pTBI. Although we observed PROX1 expression post-pTBI, further research is necessary to confirm the association between lymphangiogenesis and the post-injury response. At the sites of inflammation, macrophages, dendritic cells, neutrophils, mast cells, fibroblasts and other cells secrete a large amount of VEGF-C, VEGF-D, which enhances its binding to VEGFR-3^21^. Although inflammation is associated with lymphangiogenesis, the underlying mechanism in TBI has not been elucidated. Thus, clarifying the roles of PROX1 and VEGFR-3 in lymphangiogenesis during inflammation following TBI is a potential area that can be explored in future studies. Moreover, future follow-up research on the source of neonatal lymphatic endothelial cells following TBI is needed. We speculate that these cells may either originate from existing lymphatic vessels around the brain tissue or from the vascular endothelial cells in the brain tissue or the surrounding stem cells.

The role of PROX1 in lymphangiogenesis was previously shown in our previous studies using a sciatic nerve injury mouse model and mouse glioma model^14,22^. Our findings show the association between PROX1 expression and new lymphatic cell formation as well as the correlation between new lymphatic cell formation and damaged tissue clearance. The formation of new lymphatic cells could potentially occur through PROX1-induced differentiation of CD34-positive vascular endothelial cells around the injury site into new LYVE-1-positive lymphatic cells. This first report of new lymphatic cell formation in brain tissue after pTBI serves as a foundation for further characterization of the physiological tissue response following CNS injury.

## Methods

### Animals and grouping

Seventy male and female specific pathogen-free (SPF)-grade Kunming mice weighing 18–26 g (Laboratory Animal Center of Shandong University, Shandong, China) were housed in a sterile room at 21–23 °C under a 12 h:12 h light:dark cycle. Food and water were available ad libitum. The mice were randomly divided into seven groups (n = 10/group): normal control, 1, 3, 7, 14, 21, and 30 days after pTBI. All animal experiments conducted were in accordance with the Ethics Committee of Shandong College of Traditional Chinese Medicine (Shandong, China). All procedures performed in studies involving animals were in accordance with the ethical standards of the institution or practice at which the studies were conducted.

All animal experiments conducted are approved by the Ethics Committee of Shandong College of Traditional Chinese Medicine (Shandong, China). All procedures performed in studies involving animals were in accordance with the ethical standards of the institution or practice at which the studies were conducted.

### Establishing the mouse pTBI model

The seven groups of 70 mice were anesthetized (ketamine 50 mg/kg, mebendazole 40 mg/kg) at a dose of 2 mg/kg, and were fixed prone before cutting their heads along the midline of the brain. The focal mechanical pTBI was inflicted on all groups except the control group. The pTBI site was 1.5 mm posterior and 2.5 mm lateral to bregma and had a penetration depth of 2 cm based on a referenced protocol^23^ with modifications. Briefly, the skin on the dorsal side was separated to expose the right parietal periosteum. A 1-mL syringe with a 5 gauge needle (0.5 mm) was positioned vertically near the center of the parietal bone before piercing into the parietal lobe of the right brain to the base of the skull (pTBI site) and then pulling it out immediately. After inflicting the non-fatal pTBI to the parietal lobe of the lateral mouse brain, the skin was sutured with a No. 7 thread. As only the skin was cut and the brain was not punctured for the control group, there would be no damage to the meninges and lymphatic vessels in the cranial cavity. The mice were sacrificed at the respective timepoints postoperatively, and the brains were removed, fixed in 4% formaldehyde, and embedded in paraffin to prepare 4 μm thick sections. A total of 10 brain tissue slices per timepoint were used for visualization and analyses. The injury site could be detected in all 10 tissue slices for each timepoint, but the area of the injury site varied with the timepoint and depth of the slice. The 4 μm thick brain tissue sections were subjected to hematoxylin and eosin (H&E) staining according to a previous study^22^ to observe tissue morphological changes, and LYVE-1 and PROX1 immunohistochemical staining and LYVE-1/CD34, LYVE-1/PROX1 immunofluorescence double staining were also performed. The average integral optical density (IOD) of LYVE-1- and PROX1-positive cells/vessels was measured using an optical microscope (DP-72; Olympus, Tokyo, Japan) at 400 × magnification. Images captured were analyzed using Image-Pro Plus v6.0 software (Rockville, MD, USA).

### Immunohistochemistry

Immunohistochemical imaging was performed to observe the expression of LYVE-1 and PROX1 in the sciatic nerve. After antigen retrieval of deparaffinized mouse brain tissue sections (4 μm thick; 10 slices per timepoint) in 0.01 mol/L citrate buffer at 121 °C for 15 min, hydrogen peroxide (3 mL/L) was used to inactivate the endogenous peroxidase. The sections were first incubated with rabbit anti-mouse PROX1 antibody (1:100; Boster Biological Technology Co., Wuhan, China), rabbit anti-mouse CD34 antibody (1:100; BosterBiological Technology) or goat anti-mouse LYVE-1 antibody (1:100; R&D Systems, Shanghai, China) at 4 °C for 24 h. Next, the sections were incubated with horseradish peroxidase-labeled goat anti-rabbit IgG or rabbit anti-goat IgG secondary antibodies. DAB (3, 3-diaminobenzidine; Boster Biological Technology) immunostaining kit was subsequently used for color development (Boster Biological Technology Co.) and the tissue sections were visualized using the DP-72 optical microscope (Olympus) at 400 × magnification. Images of 50 random points per stained section were captured. Image-Pro Plus 6.0 software (Media Cybernetics, Rockville, MD) was used for semi-quantitative analysis and the IOD was calculated.

### Immunofluorescence double staining

After antigen retrieval, the paraffinized mouse brain tissue sections (4 μm thick; 10 slices per timepoint) were incubated with rabbit anti-mouse PROX1 (1:100 dilution; Boster Biological Technology) and goat anti-mouse LYVE-1 (1:100 dilution; R&D Systems) antibodies, or rabbit anti-mouse CD34 (1:100 dilution; Boster Biological Technology) and goat anti-small mouse LYVE-1 (1:100) antibodies, at 4 °C for 24 h. The sections were also incubated with Cy3-labeled donkey anti-goat IgG or FITC-labeled donkey anti-rabbit IgG (Antgene Co., Wuhan, China) before visualization and imaging with the DP-72 fluorescence microscope (Olympus).

### Statistical analysis

Statistical analysis was performed using SPSS v20 software (SPSS Inc., Chicago, IL, USA). Comparison between groups was performed using the Kruskal–Wallis H test followed by Bonferroni post-hoc analysis. *p* < 0.05 was considered statistically significant.

## Data Availability

The datasets generated and analyzed during the current study are available from the corresponding author on reasonable request.
